# Metabolomic analysis reveals the influence of HMBOX1 on RAW264.7 cells proliferation based on UPLC-MS/MS

**DOI:** 10.1186/s12864-023-09361-x

**Published:** 2023-05-19

**Authors:** Wen Jiang, Yu Jiang, Xinghai Zhang, Hongli Mu, Yuanming Song, Hengli Zhao

**Affiliations:** 1 Department of Clinical Research Center, Central Hospital Affiliated to Shandong First Medical University, Jinan, 250013 China; 2 Central Research Laboratory, the Second Hospital of Shandong University, Jinan, 250033 China

**Keywords:** HMBOX1, Macrophage, Glutamine, Metabolomics, SLC1A5

## Abstract

Macrophages are important effector cells in tumor progression and immune regulation. Previously, we demonstrated that the transcription suppressor homeobox containing 1(HMBOX1) exhibits immunosuppressive activity in LPS-induced acute liver injury by impeding macrophage infiltration and activation. We also observed a lower proliferation in HMBOX1-overexpressed RAW264.7 cells. However, the specific mechanism was unclear. Here, a work was performed to characterize HMBOX1 function related to cell proliferation from a metabolomics standpoint by comparing the metabolic profiles of HMBOX1-overexpressed RAW264.7 cells to those of the controls. Firstly, we assessed HMBOX1 anti-proliferation activity in RAW264.7 cells with CCK8 assay and clone formation. Then, we performed metabolomic analyses by ultra-liquid chromatography coupled with mass spectrometry to explore the potential mechanisms. Our results indicated that HMBOX1 inhibited the macrophage growth curve and clone formation ability. Metabolomic analyses showed significant changes in HMBOX1-overexpressed RAW264.7 metabolites. A total of 1312 metabolites were detected, and 185 differential metabolites were identified based on the criterion of OPLS-DA VIP > 1 and p value < 0.05. Kyoto Encyclopedia of Genes and Genomes (KEGG) analysis indicated that the elevated HMBOX1 in RAW264.7 inhibited the pathways of amino acid and nucleotide metabolism. Glutamine concentrations decreased significantly in HMBOX1-overexpressed macrophages, and glutamine-related transporter SLC1A5 was also downregulated. Furthermore, SLC1A5 overexpression reversed HMBOX1 inhibition of macrophage proliferation. This study demonstrated the potential mechanism of the HMBOX1/SLC1A5 pathway in cell proliferation by regulating glutamine transportation. The results may help provide a new direction for therapeutic interventions in macrophage-related inflammatory diseases.

**Supplementary Information:**

The online version contains supplementary material available at 10.1186/s12864-023-09361-x.

## Introduction

As important effector cells in tumor progression and immune regulation, macrophages are pivotal to tumorigenesis and development [1, 2]. Emerging evidence indicates that many macrophages are established from progenitors in the yolk sac and fetal liver, and then they are maintained with self-proliferation and monocyte supplementation [3–5]. Macrophage proliferation usually contributes to its accumulation in tumors or inflammatory diseases; thus, inhibiting macrophage proliferation may prevent disease progression [6, 7]. Elevated macrophage levels combined with a tumor-promoting phenotype have been linked to an undesirable outcome in patients. Unsurprisingly, macrophages have been suggested as an important target for tumor immunotherapy.

The novel human transcription suppressor homeobox containing 1 (HMBOX1) has an atypical homeobox domain containing 78 amino acids and a putative HNF1N domain [8], with the function in cell proliferation [9, 10], apoptosis [11], differentiation [12–14], and immunomodulation [15–18]. Previously, we found that HMBOX1 acts as a transcriptional repressor of interferon γ (IFN-γ) in natural killer (NK) cells [15, 16] and protects against LPS/D-GalN-induced acute liver injury by inhibiting liver inflammation. Further research identified negative regulation of NF-κB/CCL2 signal transduction in hepatocytes[17]. However, the regulatory effects of HMBOX1 on macrophage activity and proliferation have not yet been studied in detail.

As the most abundant amino acid in the human body [19], glutamine provides an energy substrate and serves as a precursor of other amino acids, including glutamic acid, aspartate, and alanine [20–23].Other important metabolites of glutamine include ammonia, lactic acid, and pyruvic acid.

Recent reviews have highlighted the importance of glutamine function in macrophages [24–26], metabolically active immune cells that require glutamine and its metabolic products for protein synthesis [27]. Moreover, glutamine metabolism profoundly influences macrophage proliferation and activation, with 10 mM glutamine reported to increase RAW264.7proliferation and viability [24, 28]. Glutamine has been shown to promote cell proliferation in general [29], acting through glutamine-related transporters of the solute carrier (SLC) family [30].

Because glutamine is hydrophilic and water-soluble [31], SLC transporter proteins (e.g., SLC1, SLC6, SLC7, and SLC38) help carry extracellular glutamine past the cell membrane [32], particularly SLC1A5 [33]. The influence of SLC1A5 on amino acid metabolism makes it an important regulator of cell proliferation [30, 34, 35]. Other SLC1A5 substrates include aspartic acid, serine, alanine, and cysteine, all of which are also involved in cell metabolism. While we observed a lower SLC1A5 levels in HMBOX1-overexpressed RAW264.7 cells, the specific influence on macrophage biological behaviour remains unclear.

This study aimed to investigate the role of HMBOX1 in regulating macrophage proliferation by using untargeted liquid chromatography coupled with mass spectrometry (LC-MS) to profile key metabolites and characterize their variation. The profiling results allowed for clarification of the mechanism underlying HMBOX1 effects on macrophage proliferation. Our data clearly showed that HMBOX1-overexpressed macrophages display distinct metabolic signatures and associated changes in relevant metabolites. Furthermore, we have provided valuable empirical evidence of HMBOX1 function in macrophage proliferation, which is expected to be a potential therapeutic target in the treatment of tumors and inflammatory diseases.

## Methods

### Cell lines and cell culture

Murine macrophage cell line RAW264.7 was obtained from the Cell Bank of Type Culture Collection at the Chinese Academy of Sciences (Shanghai, China). All cells were maintained in DMEM (Gibco BRL, Grand Island, NY, USA) supplemented with 10% fetal bovine serum (FBS) (Sijiqing, Hangzhou, China).

### Cell transfection

RAW264.7 cells were plated at a density of 2 × 10^5^ cells/well in 6 plates and transfected with HMBOX1-overexpression plasmid (pcDNA3.1-HMBOX1 plasmid, YouBia, China) or blank vector for 6 h, and they were then cultured at 37 ℃ for 24 h. As for lentivirus transduction, RAW264.7 cells were plated onto six-well plates and transduced with lentivirus supernatants containing HMBOX1-overexpressed plasmid (pHBLV-CMV-MCS-fLUC-EF1-ZsGreen-T2A-PURO, Hanbio, China) for 8 h. After washing twice with phosphate-buffered saline (PBS), cells were cultured in DMEM for 48 h, after which they were cultured with 1.0 µg/mL puromycin (Sigma-Aldrich, St. Louis, MO, USA) for 5 days before use. The HMBOX1-overexpressed RAW264.7 cells were plated at density of 2 × 10^5^ cells/well in 6 plates and transfected with SLC1A5-overexpression plasmid (pCDH3.1-CMV-MCS-EF1-Neo, Keyybio, China) or blank vector for 6 h; then, they were cultured at 37 ℃ for 24 h. Cells were then collected for the subsequent experiments.

### Metabolite extraction

HMBOX1-lentiviral plasmid transducted RAW264.7 were incubated with LPS (10 ng/mL) for 6 h. After washing with 3 mL PBS (Sigma-Aldrich) at 37 °C, cells were extracted (1 mL per 5 × 10^6^ cells) using an ice-cold solution (methanol-acetonitrile-water = 40:40:20, v/v). Next, cells were treated with ultrasound for 30 min at 4 °C and centrifuged for 20 min at 14,000 × g. The supernatant was collected, vacuum-dried, redissolved with acetonitrile-water solution (1:1,v/v), and transferred into a high-performance liquid chromatography (HPLC) vial.

### LC-MS conditions

We performed LC-MS analysis with the Agilent 1290 Infinity UHPLC system interfaced with an AB Triple TOF 6600 (AB Sciex, Germany) and HILIC (150 × 4.6 mm, 5 μm) HPLC columns (Hi Chrom; Reading, UK). The HILIC mobile phase consisted of 25 mM ammonium acetate and 20 ammonia water in (A) HPLC-grade water and (B) acetonitrile. The solvent gradient was 95% B (0–0.5 min), 95–65% B (0.5–7 min), 65–40% B (7–8 min), and 40–95% B (9.1–12 min); it was then maintained at 95%, and the flow rate was 0.5 mL/min. Nitrogen sheath and auxiliary gas flow rates were maintained at 30 and 60 arbitrary units, respectively. The electrospray ionization interface was set to the positive/negative dual-polarity mode with a spray voltage of 5.5 kV and ion transfer capillary temperature of600°C. Full-scan data were obtained under a mass-to‐charge ratio (m/z) between 25 and 1000 amu for both ionization modes. To monitor stability and repeatability, quality control (QC) samples were prepared by pooling 10 µL of each sample. The QC samples were inserted after every five regular samples.

### Data extraction and processing

Data were extracted in XCMS Online. Isotopes and adducts were annotated with Collection of Algorithms of Metabolite pRofile Annotation (CAMERA). Peaks of the resultant metabolite lists were then manually evaluated. Suitable metabolites were matched with retention times of authentic standard mixtures run in the same sequences. Metabolite identification was validated with library searches against accurate metabolite-mass data from the Human Metabolome Database and Kyoto Encyclopedia of Genes and Genomes (KEGG) [36, 37].

### Statistical analysis

Data were subjected to multivariate Pareto-scaled principal component analysis (PCA) and orthogonal partial least-squares discriminant analysis (OPLS-DA) using the ropls R package. Model robustness was evaluated with seven-fold cross-validation and response permutation testing. Variable importance in the projection (VIP) for each component of the OPLS-DA model was calculated to determine its contribution to classification. Independent Student’s t-tests were used to determine between-group differences. Metabolites were considered significantly changed if VIP > 1 and p < 0.05. Relationships between variables were assessed with Pearson’s correlation analyses.

## Results

### HMBOX1 effects on RAW264.7 proliferation

**Fig. 1:**
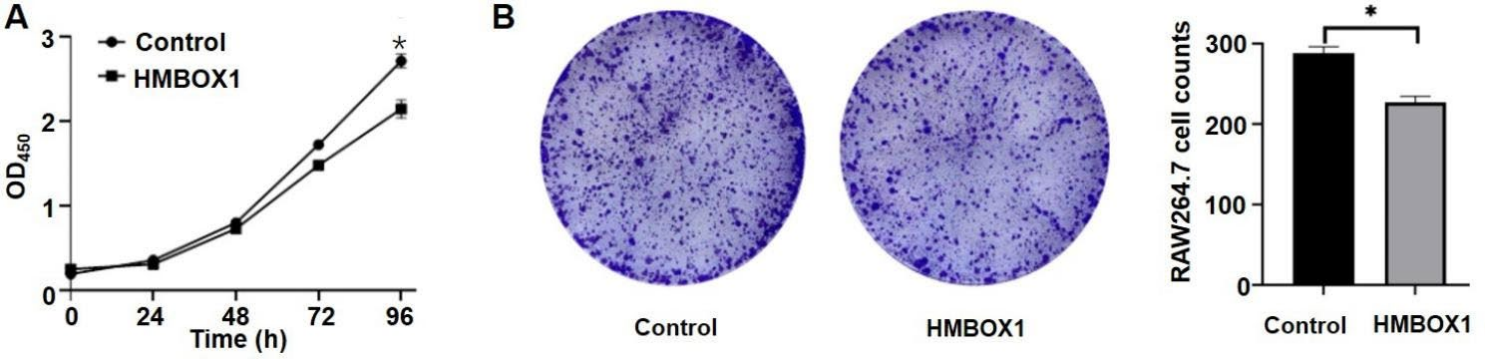
HMBOX1 inhibited macrophage proliferation. RAW264.7 cells transfected with HMBOX1-lentiviral or the controls were stimulated with LPS (10 ng/mL) for 6 h, and (**A**) CCK8 assay and (**B**) clone formation assay were performed to evaluate the cells proliferation ability. Each value represented the mean ± SD of triplicate tests. *p < 0.05

RAW264.7 cells were transduced with HMBOX1-overexpressed lentivirus plasmid, increased HMBOX1 level was observed (Fig. S1a). The cells were collected for CCK8 assays at 24, 48, 72, and 96 h, and the results revealed decreased proliferation in RAW264.7 cells (Fig. 1a). A clone formation assay yielded similar outcomes (Fig. 1b). These results suggest that HMBOX1 has a significant effect on RAW264.7 cell proliferation.

### HMBOX1 effects on LPS-induced RAW264.7 activation

**Fig. 2:**
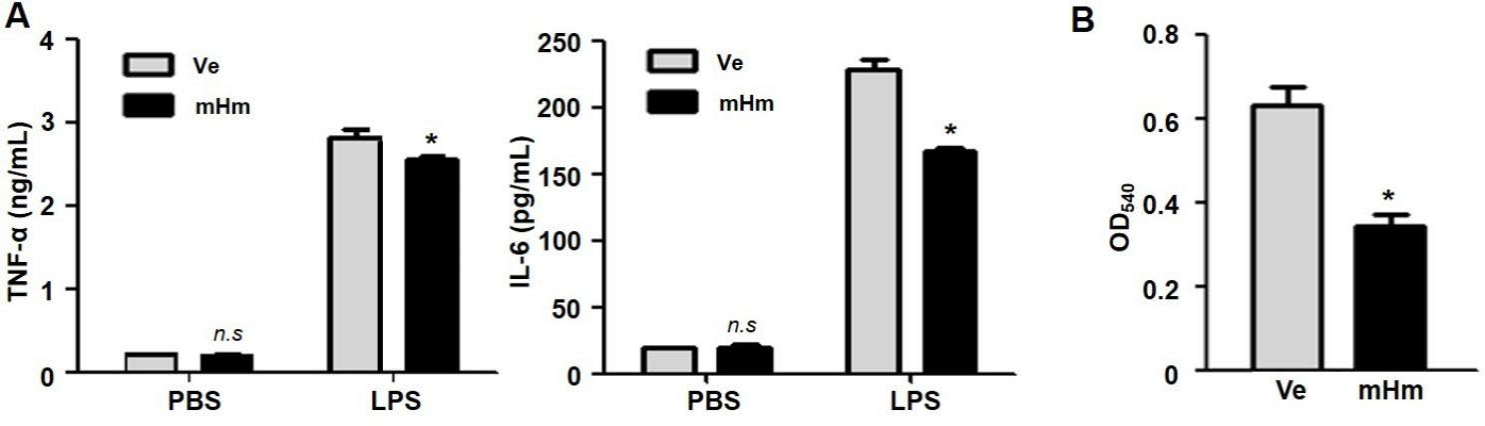
HMBOX1 affected LPS-induced macrophage activation. Transfected RAW264.7 cells were plated at density of 2 × 10^5^ cells/well in 6 plates with 2 mL DMEM and stimulated with LPS (10 ng/mL). (**A**) Decreased levels of cytokines TNF-α and IL-6 were observed in the HMBOX1-overexpressed group with 24 h LPS stimulation. (**B**) RAW264.7 cells were stimulated with LPS (10 ng/mL) for 3 h, then, neutral red solution (0.1%) was added and co-incubated with RAW264.7 cells for 1 h. Decreased phagocytosis ability was also observed in the HMBOX1-overexpressed RAW264.7 cells with neutral red assay. Statistical significance was determined as *, p < 0.05 and **, p < 0.01 compared with the controls and represents the mean ± SD of triplicate tests. mHm: pcDNA3.1-HMBOX1 plasmid; Ve: pcDNA3.1 plasmid

Transfected with pcDNA3.1-HMBOX1 plasmid (Fig. S1b) or the controls, RAW264.7 cells were stimulated with LPS (10 ng/mL) for 24 h. There were significant decreases in the levels of TNF-α and IL-6 in LPS-stimulated RAW264.7 cell supernatant compared to those in the control group (Fig. 2a). Meanwhile, impaired phagocytosis ability was observed in the HMBOX1-overexpressed RAW264.7 cells compared to those in the controls, with a lower percentage of neutral-red-positive macrophages (Fig. 2b). These results indicate that HMBOX1 has a significant effect on RAW264.7 cell activation.

### Differential metabolites profiles between the control and HMBOX1-overexpressed groups

**Fig. 3:**
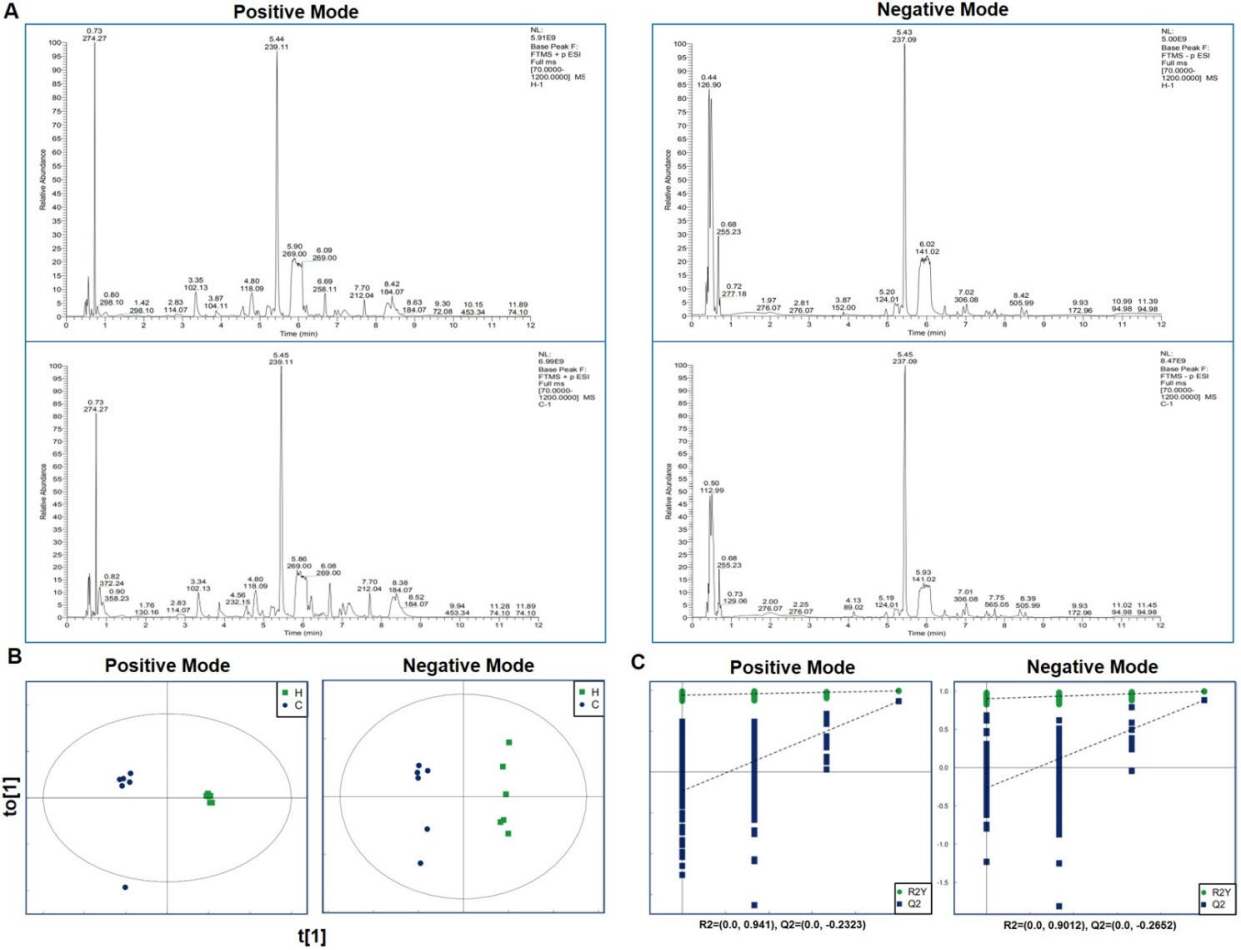
HMBOX1 affected macrophage metabolite profiles. (**A**) Total ion flow charts of cell metabolites in the control group and HMBOX1-overexpressed group (n = 6). (**B**) OPLS-DA score map and (**C**) results of the OPLS-DA confidence test. C: the control group; H: HMBOX1-overexpressed group

As shown in Fig. 3a, the total ion chromatograph (TIC) of cell metabolites in the control and HMBOX1-overexpressed groups were identified to bein the HILIC positive or negative ion mode. On this basis, we initially used Compound Discoverer software to process data and obtain a matrix which included 1312 metabolites, including lipids, nucleotides, organic acids, and organic nitrogen. The data obtained were exported into R package for multivariate statistical analysis using the PLS-DA, PCA, and OPLS-DA models. The OPLS-DA score scatterplot showed a clear distinction between the two groups (Fig. 3b). The confidence test result of the OPLS-DA model also indicated that there was no overfitting phenomenon in the OPLS-DA model (Fig. 3c).

### Identification of differential metabolites in the control and HMBOX1-overexpressed groups

**Fig. 4:**
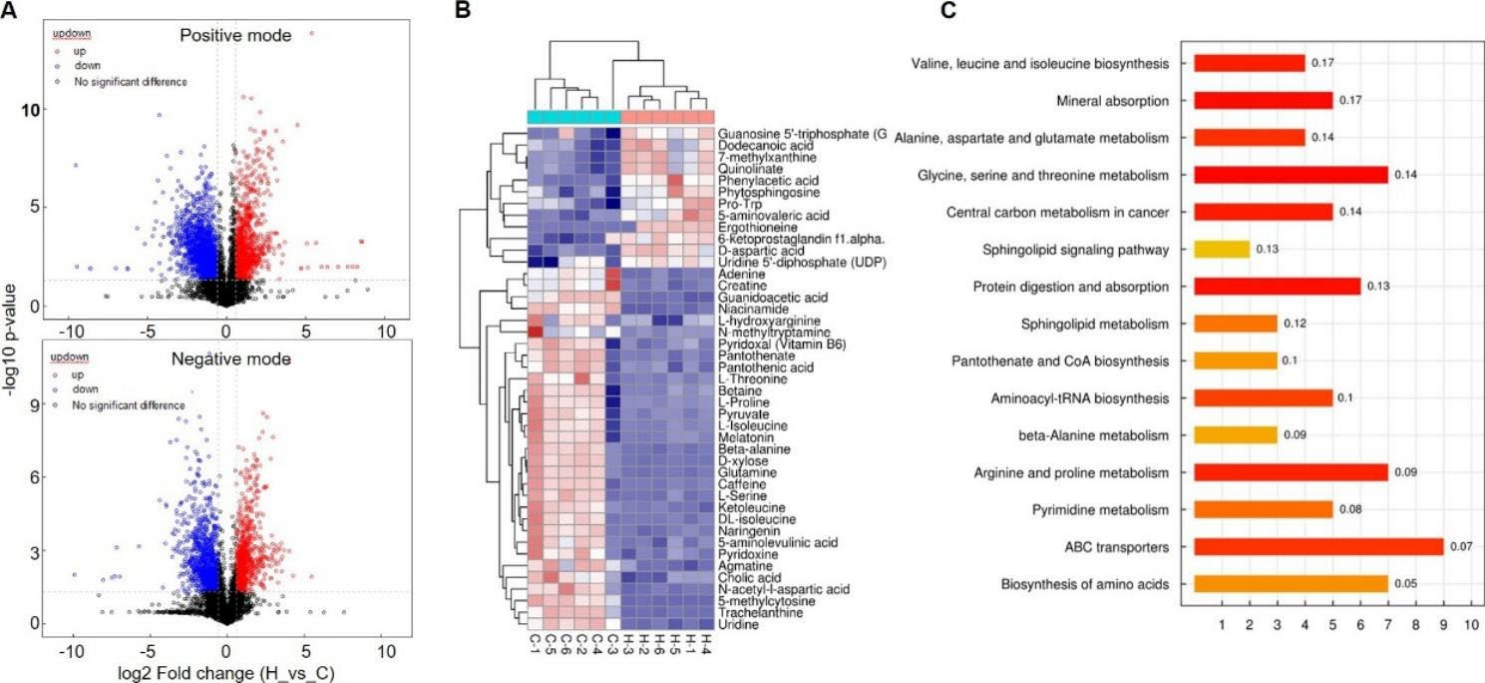
HMBOX1 inhibited amino acid and nucleotide metabolism in RAW264.7 cells. (**A**) Volcano plot analysis for the differential metabolites in RAW264.7 cells transfected with HMBOX1 lentiviral and the controls. (n = 6). (**B**) KEGG heatmap analysis on differential metabolites in RAW264.7 cells (differential metabolites counts > 5). (**C**) Overview of metabolite enrichment in RAW264.7 cells

As shown in Fig. 4a, the volcano plot indicated that HMBOX1-overexpressed macrophages had significantly different metabolite expression (fold change [FC] > 1.5 or < 0.67 and p < 0.05). The results of KEGG analysis on differential metabolites revealed that they participated in the same metabolic processes or cellular pathways (heatmap, Fig. 4b). Additionally, HMBOX1 overexpression affected multiple types of amino acid and nucleotide metabolism (Fig. 4c), such as alanine, aspartate and glutamate metabolism, arginine and proline metabolism, glycine, serine and threonine metabolism, valine, leucine and isoleucine biosynthesis, and pyrimidine metabolism. The differential metabolites in amino acid and nucleotide metabolism are listed in Table 1. These results collectively suggest the regulatory function of HMBOX1 in RAW264.7 cell metabolism.

**Table 1:** Information of differential metabolites in HMBOX1-overexpressed RAW264.7 in response to LPS.

Compound	Fold change	p-value	m/z	rt(s)	Adduct	Ionisation Mode
L-Serine	0.467	0.001	104.035	318.322	(M-H)^−^	−
L-Threonine	0.474	0.010	118.051	301.351	(M-H)^−^	−
Pyruvate	0.380	0.015	87.009	121.803	(M-H)^−^	−
Guanidoacetic acid	0.407	0.000	118.061	361.471	[M + H]^+^	+
Creatine	0.555	0.002	154.059	356.294	[M + Na]^+^	+
5-aminolevulinic acid	0.711	0.002	114.066	172.044	[M + H-H_2_O]^+^	+
Betaine	0.785	0.031	118.086	286.827	[M + H]^+^	+
Ketoleucine	0.284	0.001	129.056	43.912	[M-H]^−^	−
L-Isoleucine	0.356	0.014	130.087	247.704	[M-H]^−^	−
DL-isoleucine	0.447	0.002	132.102	286.146	[M + H]^+^	+
D-aspartic acid	1.749	0.000	132.030	424.707	[M-H]^−^	−
N-acetyl-l-aspartic acid	0.708	0.000	176.041	356.294	[M + H]^+^	+
Glutamine	0.429	0.000	147.076	372.540	[M + H]^+^	+
L-Proline	0.493	0.036	114.056	270.866	[M-H]^−^	−
Agmatine	0.344	0.000	131.118	421.243	[M + H]^+^	+
L-hydroxyarginine	0.545	0.037	116.071	331.547	[M + H-CH_5_ON_3_]^+^	+
Pantothenate	0.494	0.002	218.103	247.003	[M-H]^−^	−
Beta-alanine	0.665	0.000	90.055	366.517	[M + H]^+^	+
Quinolinate	3.131	0.000	150.022	127.883	[M + H-H_2_O]^+^	+
Pantothenic acid	0.620	0.007	220.118	291.144	[M + H]^+^	+
Uridine	0.339	0.000	267.065	288.039	[M + Na]^+^	+
5-methylcytosine	0.200	0.000	126.066	46.761	[M + H]^+^	+
Uridine 5’-diphosphate (UDP)	1.827	0.028	405.009	419.068	[M + H]^+^	+
Phytosphingosine	1.454	0.003	318.300	44.625	[M + H]^+^	+
Pro-Trp	1.302	0.008	302.305	44.102	[M + H]^+^	+

### HMBOX1 downregulated glutamine level in RAW264.7 cells by inhibiting SLC1A5-mediated intracellular transportation

**Fig. 5:**
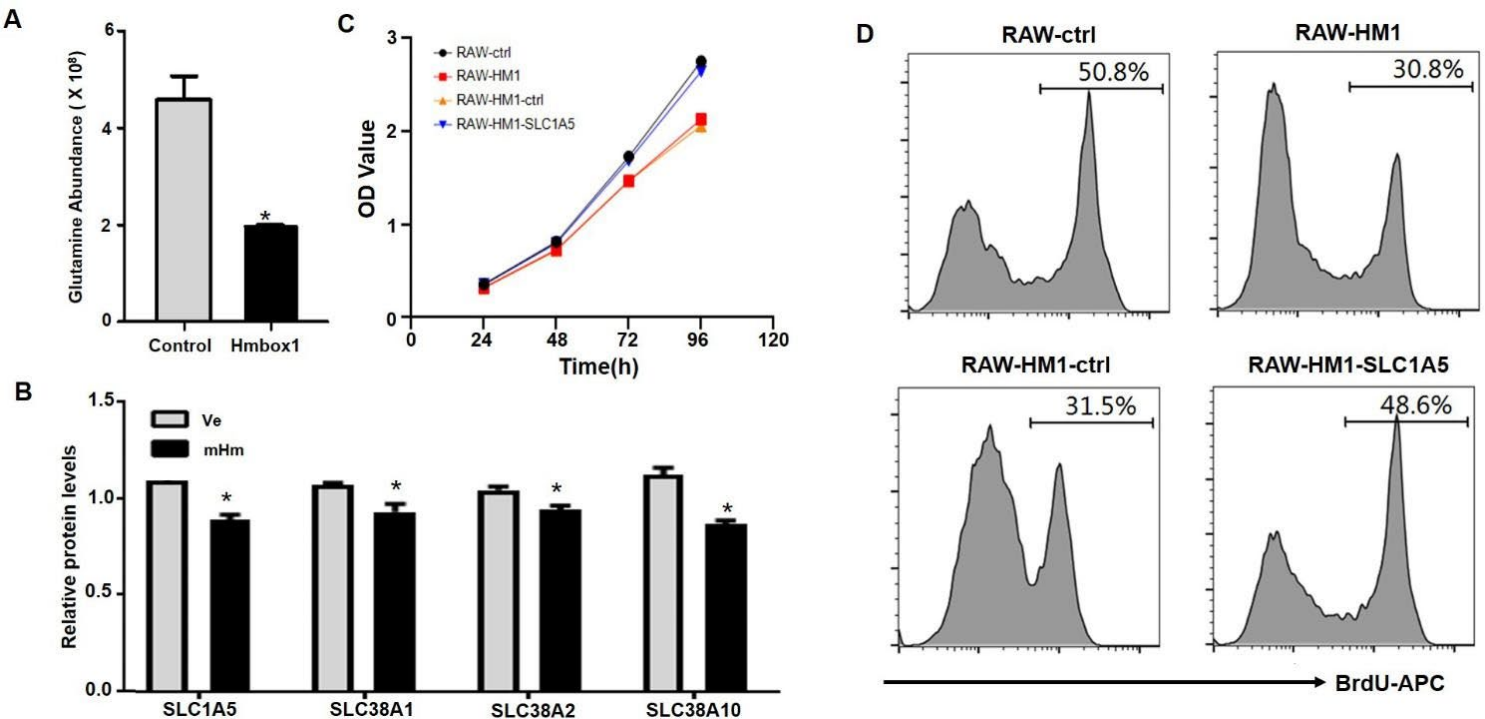
HMBOX1 downregulated glutamine level in RAW264.7 cells by inhibiting Slc-mediated intracellular transportation. RAW264.7 cells were transfected with HMBOX1 lentiviral or the controls and stimulated with 10 ng/mL LPS for 6 h. Decreased levels of (**A**) glutamine and (**B**) glutamine-related transporters were observed in macrophages following HMBOX overexpression. (**C**) SLC1A5 overexpression partially recovered the proliferation ability of RAW264.7 cells transfected with HMBOX1 lentiviral with CCK8 assay, as well as the (D) BrdU-positive RAW264.7 cells determined by flow cytometry

As shown in Table 1, several amino acid metabolites were significantly decreased following HMBOX1 overexpression, these metabolites have important roles in cell proliferation and biological function. Glutamine, for instance, serves as a precursor of other amino acids and is a key amino acid for macrophage proliferation and activation. HMBOX1-overexpressed RAW264.7 cells had lower glutamine levels than the control cells (Fig. 5a). Additionally, glutamine-related transporters, including SLC1A5, SLC38A1, SLC38A2, and SLC38A10, were observed to be negatively regulated by the overexpressed HMBOX1, using the LC-MS/MS method (Fig. 5b). Thus, the mechanism of HMBOX1 inhibition of macrophage proliferation appears to involve the downregulation of glutamine transporters and the limiting of glutamine intracellular transport.

Previous research reported that cancer cells took up glutamine through Slc family members, including SLC1, 6, 7, and 38 [38]. Among them, SLC1A5 has been deeply studied and acted as an obligatory sodium-dependent transporter for neutral amino acids [39]. SLC1A5 inhibition impaired glutamine uptake and cell multiplication capacity [40]. To confirm SLC1A5 participation in the regulatory function of HMBOX1 in cell proliferation, we overexpressed SLC1A5 in RAW264.7 cells transducted with HMBOX1 lentiviral plasmid (Fig. S1c). A CCK8 proliferation experiment identified that SLC1A5 overexpression significantly reversed HMBOX1 inhibition on cell proliferation (Fig. 5c). Our BrdU proliferation experiment confirmed these findings, along with the restored proliferation ability of HMBOX1-overexpressed RAW264.7 cells (Fig. 5d). These results suggest that SLC1A5-mediated glutamine transport plays a major role in RAW264.7 cell proliferation and that HMBOX1/SLC1A5 is a potential target for regulating macrophage proliferation.

## Discussion

Although it is a fairly novel field, metabolomics has already made important contributions to research on macrophage function [41–43]. Here, we applied metabolomics to demonstrate that transcription repressor HMBOX1 downregulated intracellular glutamine levels to block macrophage proliferation. Proteomics and in vitro experiments indicated that the mechanism involved HMBOX1 inhibiting the expression of SLC glutamine-related transporters. These results show that HMBOX1 has an important regulatory role in macrophage-related immune diseases.

Our metabolomics analysis also revealed many other amino acids that were significantly inhibited by HMBOX1, including valine, leucine, isoleucine, glycine, serine, threonine, arginine, proline, and pyrimidine. These effects are likely attributable to the importance of glutamine metabolism as a source of material for synthesizing other intracellular components. For instance, amide nitrogen in glutamine is necessary for biosynthesis, and cells in vivo use glutamine to produce purines, pyrimidines, and other amino acids [22, 23, 44, 45]. To explore the mechanism of glutamine effects on macrophage proliferation, we cultured HMBOX1-overexpressed RAW264.7 cells in DMEM culture medium supplemented with 2mmol/L L-glutamine. However, the inhibition function of HMBOX1 on cell proliferation was not improved by the increased L-glutamine in medium (Fig. S2), suggesting that the level of glutamine in the extracellular medium was sufficient for cell growth, and the inhibition of HMBOX1 on the proliferation of RAW264.7 cells was mainly related to its suppression of the intracellular transport of glutamine, but not the level of extracellular glutamine possibly.

In our study, overexpressed HMBOX1 down-regulated the level of macrophage glutamine transporter SLC1A5. We also observed decreased glutamine level in macrophages, as well as the down-regulate metabolism of various amino acids and pyrimidine nucleotides, which was consistent with the previous reports. All of the data suggested the mechanism of HMBOX1 in inhibiting cell multiplication capacity by targeting SLC1A5-related glutamine transportation. Further study was needed to disclose the specific regulatory mechanism. Additionally, our proteomics analyses showed that HMBOX1 suppressed the expression of glutamine transporter and glutaminase (with 10% suppression) but not that of glutamine synthetase or glutamine dehydrogenase. Therefore, HMBOX1 seems to be important for glutamine transportation but not for its metabolism.

Except for the SLC family transporters, the phytosphingosine level was significantly promoted following HMBOX1 overexpression. It has been reported that phytosphingosine induces cell apoptosis via a mitochondrially mediated pathway [46],which maybe another mechanism for HMBOX1function in inhibiting RAW264.7 proliferation ability. It may also be a reason for the partial recovery of cell proliferation after SLC1A5 was elevated.

In addition to promoting cell proliferation, glutamine supports macrophage immune function, including phagocytosis, pro-inflammatory cytokine synthesis/secretion, and antigen presentation [47, 48]. Jiang et al. reported that glutamine serves as a carbon and nitrogen source for the metabolic reprogramming to M1-like macrophages [49]. Our study demonstrated that HMBOX1 significantly inhibits LPS-induced M1 activation of macrophages, with decreased M1-related cytokines and cell phagocytic ability. Evidence from this study indicates that HMBOX1 suppression of glutamine intracellular transportation is an important contributor. Therefore, HMBOX1/SLC1A5-mediated downregulation of glutamine uptake may be one mechanism for the protective effects of HMBOX1 in liver inflammation.

Elevated HMBOX1 has also been shown to influence sphingomyelin metabolism in macrophages;the lipid sphingomyelin accounts for approximately 25% of macrophage membranes [50], participating in phagocytosis, lysosome stabilization, receptor-mediated chemotaxis, antigen presentation [51–53], and regulation of TLR4-mediated innate immune responses [54, 55]. Thus, sphingomyelin metabolism may be another pathway that HMBOX1 acts upon to regulate macrophage immune function. However, the mechanism is not yet clear, and further investigation is required to confirm this hypothesis.

## Conclusions

Our results demonstrate that HMBOX1 inhibits the proliferation and M1 activation of RAW264.7 cells by modulating the levels of many metabolites and corresponding metabolic pathways in RAW264.7 cells. Downregulated glutamine, mediated by HMBOX1-inhibited SLC1A5 function affected RAW264.7 cell proliferation and biological function. These data support our previous work on HMBOX1 inhibition function on macrophage proliferation and M1 activation. The results obtained in the present study provide the foundation for further exploration of HMBOX1 function in macrophage biological function.

## Electronic supplementary material

Below is the link to the electronic supplementary material.

Supplementary Material 1

## Data Availability

The datasets used and/or analysed during the current study available from the corresponding author on reasonable request.
