# Supplementary material for: Metabolomic analysis reveals the influence of HMBOX1 on RAW264.7 cells proliferation based on UPLC-MS/MS
Source: BMC Genomics. 2023 May 19;24:272. doi: 10.1186/s12864-023-09361-x (PMC10199573; doi:10.1186/s12864-023-09361-x)
Supplement: Supplementary file 1 — Supplementary Material 1 [file 12864_2023_9361_MOESM1_ESM.docx]

**Supplementary materials**

**Quantitative real-time PCR analysis**

Total RNA was extracted with TRIzol (Invitrogen, Carlsbad, CA, USA) for cDNA synthesis using M-MLV (Invitrogen), following the manufacturer’s protocol. Specific transcripts were detected via real-time PCR with SYBR Green Master Mix (Toyobo, Osaka, Japan) in the iCycleriQ real-time PCR system (Bio-Rad, Hercules, CA, USA). Relative expression was normalized to β-actin.


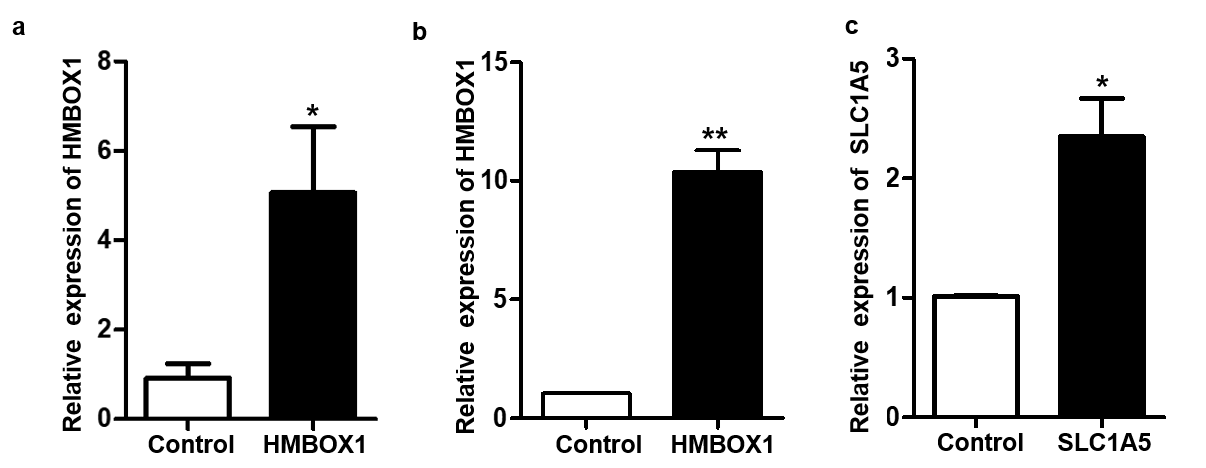


**Supplementary Figure 1.** RAW264.7 cells were transduced with lentivirus supernatants containing HMBOX1-overexpressed plasmid(a) or pcDNA3.1-HMBOX1 plasmid (b), increased HMBOX1 mRNA levels were observed. (c) HMBOX1-overexpressed RAW264.7 cells were transfected with SLC1A5-overexpression plasmid, increased SLC1A5 mRNA level was observed. Statistical significance was determined as *, p < 0.05 and **, p < 0.01 compared with the controls and represents the mean ± SD of triplicate tests. HMBOX1: HMBOX1 overexpression; SLC1A5: SLC1A5 overexpression.


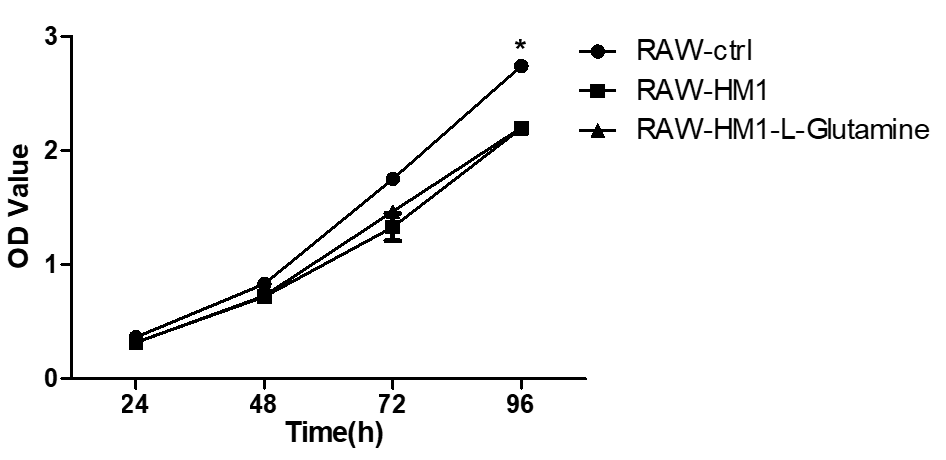


**Supplementary Figure 2.** **Extracellular glutamine increasing did not affect the inhibitory effect of HMBOX1 on macrophage proliferation ability.** RAW264.7 cells were transduced with lentivirus supernatants containing HMBOX1-overexpressed plasmid (RAW-HM1) or the controls (RAW-ctrl), then the cells were cultured with the normal culture medium added 2 mmol/L L-Glutamine (RAW-HM1-Glutamine) or the controls (RAW-ctrl and RAW-HM1) for 24h, 48h, 72h and 96h. Statistical significance was determined as *, p < 0.05 compared with RAW-ctrl group. HM1: HMBOX1 overexpression.
